# Supplementary material for: Accurate Prediction of Protein Structural Flexibility by Deep Learning Integrating Intricate Atomic Structures and Cryo-EM Density Information
Source: Nat Commun. 2024 Jul 2;15:5538. doi: 10.1038/s41467-024-49858-x (PMC11219796; doi:10.1038/s41467-024-49858-x)
Supplement: Supplementary file 1 — Supplementary Information [file 41467_2024_49858_MOESM1_ESM.pdf]

RMSF-net: Accurate Prediction of Protein Structural Flexibility by Integrating Intricate Atomic Structures and Cryo-EM Density Information

Xintao Song[1,2,3,†], Lei Bao[4,†], Chenjie Feng[5], Qiang Huang[1], Fa Zhang[6,*], Xin Gao[3,*], Renmin Han[1,2,*]

[1]Research Center for Mathematics and Interdisciplinary Sciences (Ministry of Education Frontiers Science Center for Nonlinear Expectations), Shandong University, Qingdao 266237, China; [2]BioMap Research, Menlo Park, CA 94025, USA; [3]King Abdullah University of Science and Technology (KAUST), Computational Bioscience Research Center (CBRC), Computer, Electrical and Mathematical Sciences and Engineering (CEMSE) Division, Thuwal, 23955, Saudi Arabia; [4]School of Public Health, Hubei University of Medicine, Shiyan 442000, China; [5]College of Medical Information and Engineering, Ningxia Medical University, Yinchuan 750004, China; [6] School of Medical Technology, Beijing Institute of Technology, Beijing 100081, China.

[†]These authors should be regarded as Joint First Authors; [*]All correspondence should be addressed to Fa Zhang (zhangfa@bit.edu.cn), Xin Gao (xin.gao@kaust.edu.sa) and Renmin Han (hanrenmin@sdu.edu.cn).

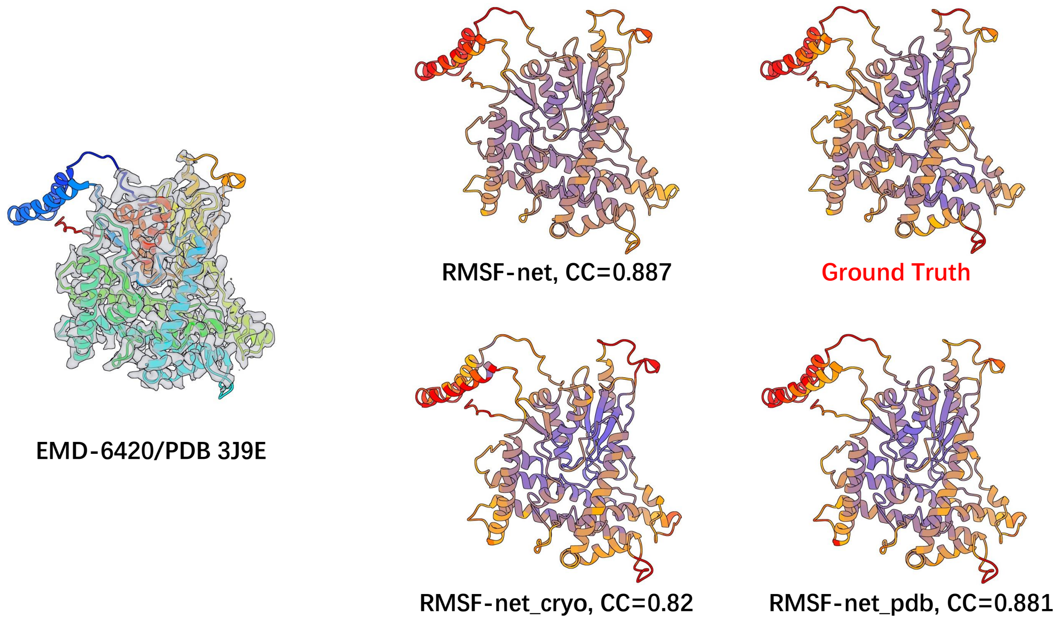

Figure S1: **RMSF predictions on EMD-6240/PDB 3J9E by RMSF-net, RMSF-net_cryo and RMSF-net_pdb along with the ground truth.**

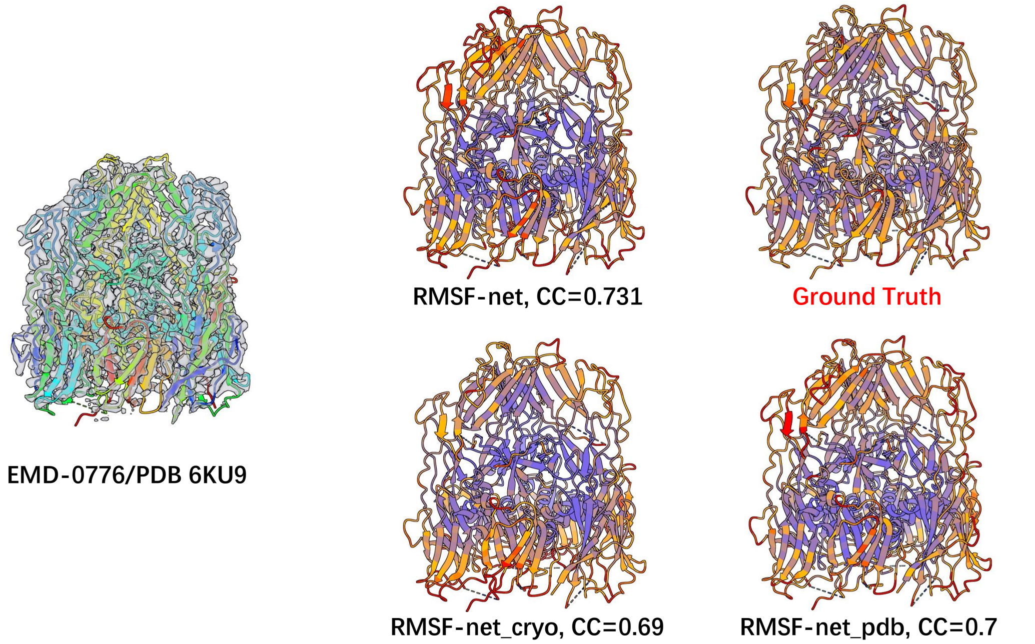

Figure S2: **RMSF predictions on EMD-0776/PDB 6KU9 by RMSF-net, RMSF-net_cryo and RMSF-net_pdb along with the ground truth.**

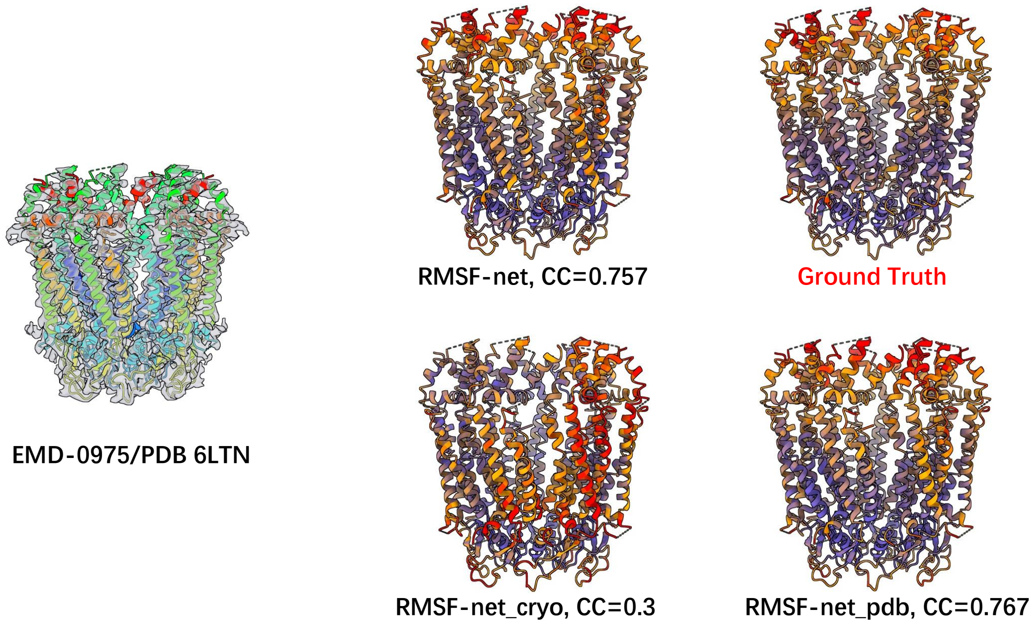

Figure S3: **RMSF predictions on EMD-0975/PDB 6LTN by RMSF-net, RMSF-net_cryo and RMSF-net_pdb along with the ground truth.**

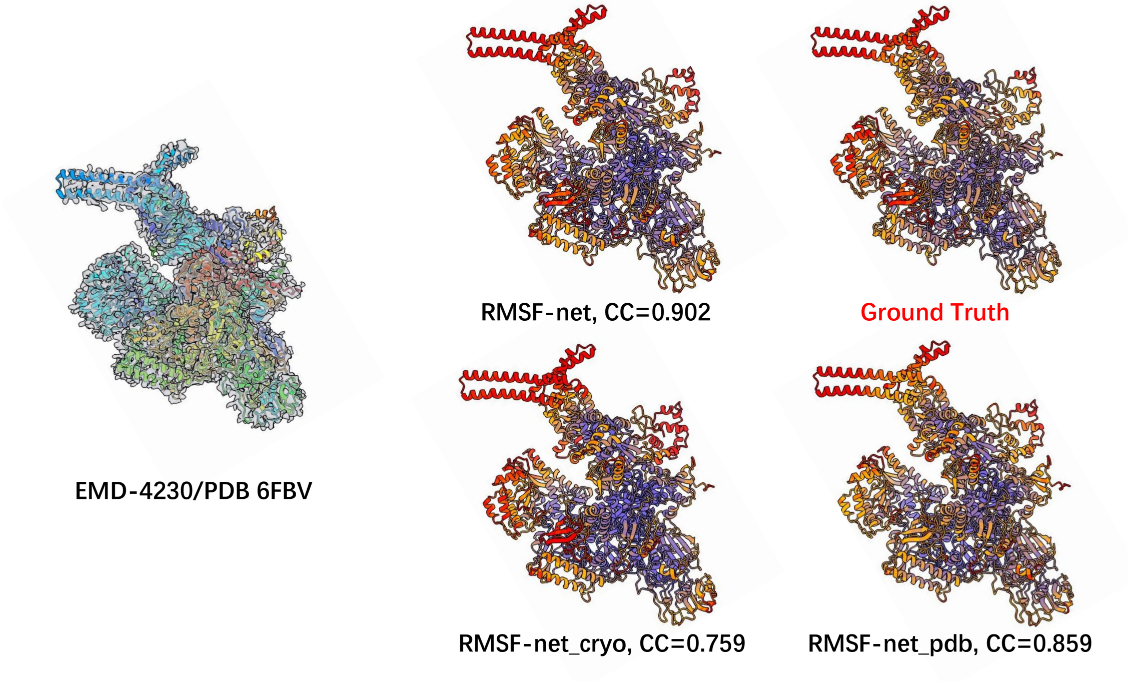

Figure S4: **RMSF predictions on EMD-4230/PDB 6FBV by RMSF-net, RMSF-net_cryo and RMSF-net_pdb along with the ground truth.**

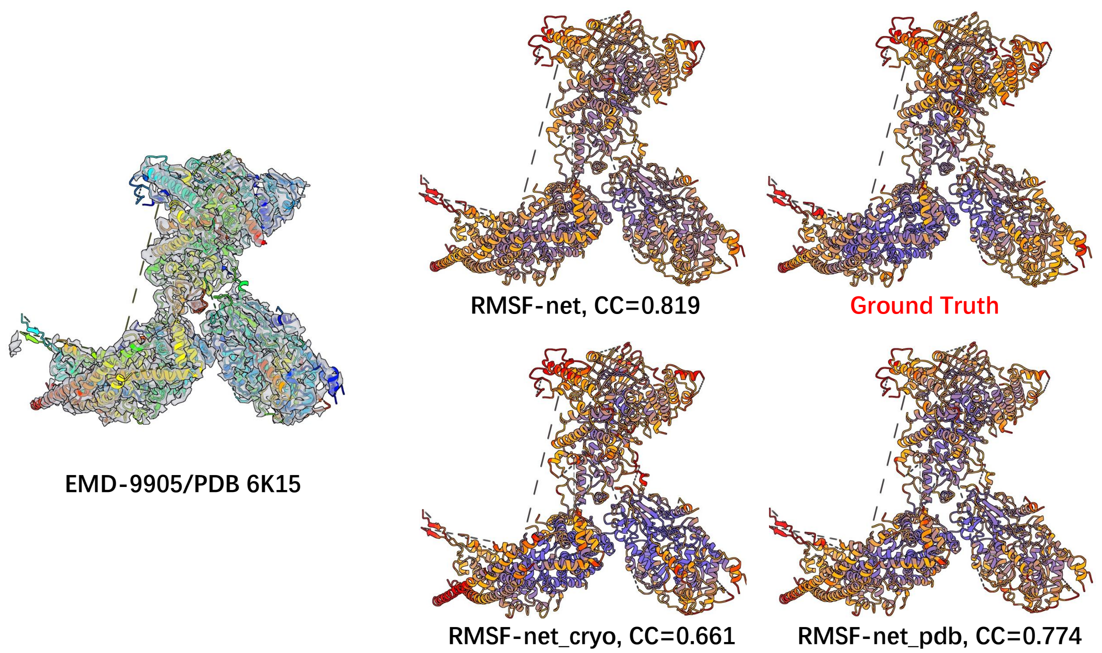

Figure S5: **RMSF predictions on EMD-9905/PDB 6K15 by RMSF-net, RMSF-net_cryo and RMSF-net_pdb along with the ground truth.**

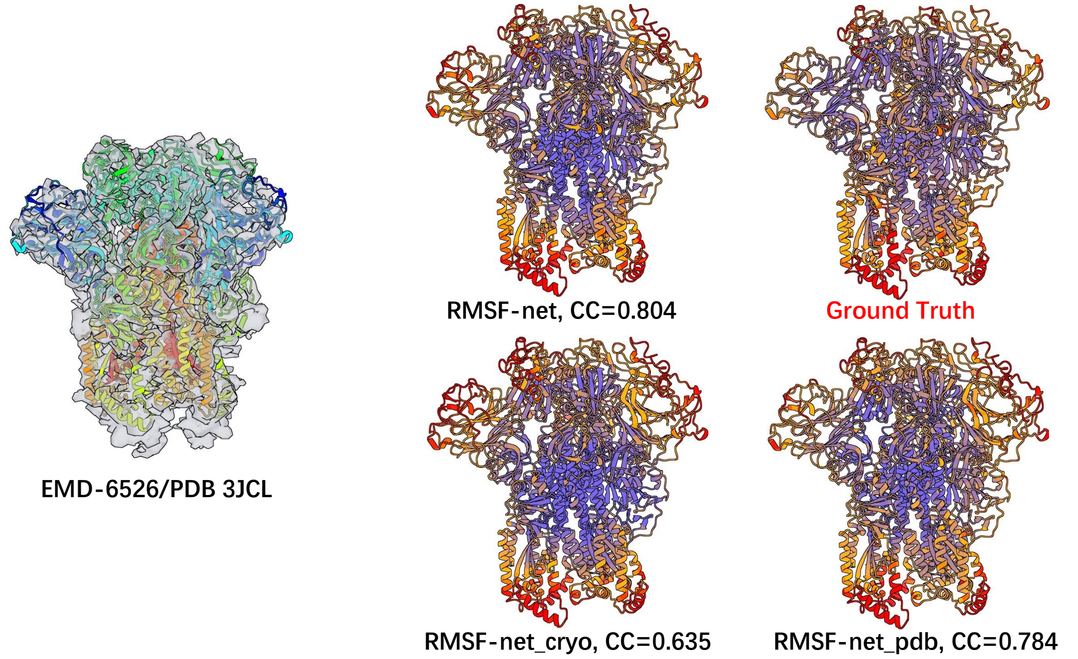

Figure S6: **RMSF predictions on EMD-6526/PDB 3JCL by RMSF-net, RMSF-net_cryo and RMSF-net_pdb along with the ground truth.**

Structure of Occ-net and the data annotation process

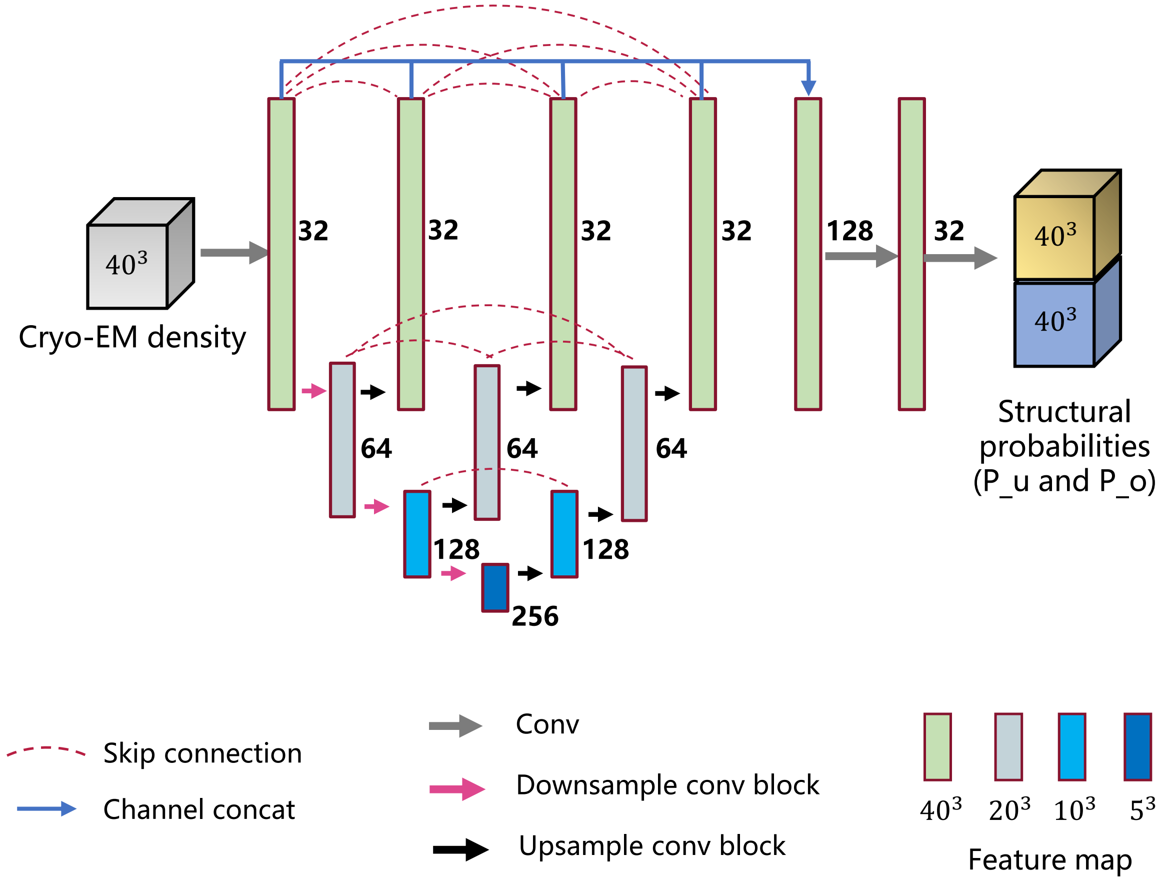

Figure S7: **The structure of Occ-net.** Occ-net serves as a structure segmentation model similar to models in other related works, such as Haruspex (Mostosi *et al.*, 2020) and EMNUSS (He and Huang, 2021). Like RMSF-net, Occ-net also adopts Unet++(L3) for structure segmentation on cryo-EM maps. The cryo-EM density (40^3) are input into Occ-net through encodings and decodings by 3D convolution blocks, and the two-channel structure probability map is output(40^3). One of the channels, P_o, corresponds to the probabilities that the voxels are occupied by the structure. The other P_u corresponds to the probabilities that the voxels are in the structure-free regions.

The PDB simulated maps corresponding to the cryo-EM maps are initially annotated to generate structure labels for training and evaluating Occ-net, where voxels with density higher than 3 σ (σ represents r.m.s.d of the PDB simluated map density) are labeled as positive class; otherwise, they are labeled as negative. Then, based on the spatial correspondence between the cryo-EM map and the PDB simulated map, class labels are annotated onto the cryo-EM maps, obtaining the structure annotation maps corresponding to the cryo-EM maps.

MD simulations over longer time periods

We conducted MD simulations for hundreds of nanoseconds on three proteins in the dataset to ascertain the robustness of the structural fluctuations obtained from the 30 ns simulation, including the cryo-EM structure of a coronavirus spike glycoprotein trimer (Walls *et al.*, 2016) (PDB 3JCL), the Human Adenovirus type 3 fiber knob in complex with one copy of Desmoglein-2 (Vassal-Stermann *et al.*, 2019) (PDB 6QNT), and the XPF-ERCC1 cryo-EM structure, apo-form (Jones *et al.*, 2020) (PDB 6SXA). We initially performed a 200 ns simulation for 3JCL, keeping all the settings the same as before except for extending the final production run time. Following the simulation, we computed the RMSF and compared it with the previous 30 ns simulation; the corresponding scatter plots and visualizations are provided in Figure S8. The results reveal a high correlation of 0.811 between the new and the old simulations. Furthermore, the predicted values of RMSF-net exhibit a correlation of 0.819 with the 200 ns MD simulation, which is even higher than the correlation of 0.804 with the original 30 ns. Subsequently, we conducted simulations lasting

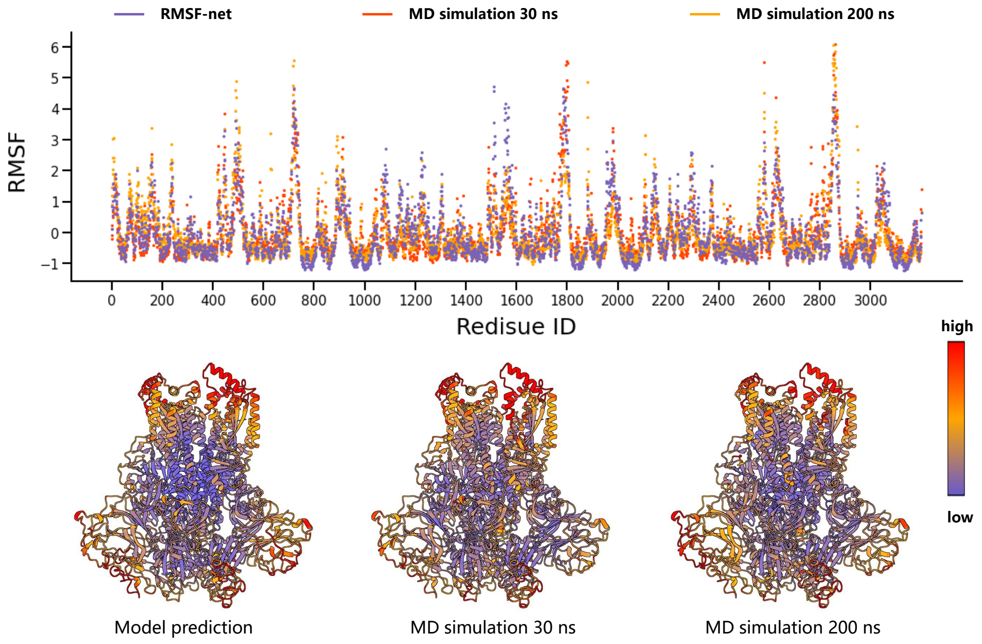

Figure S8: **Comparisons of RMSF obtained from MD simulations for 30 ns, 200 ns, and RMSF-net on 3JCL.** The first column shows scatter plots of RMSF on residues, with colors corresponding to the three approaches indicated in the legend. The second column presents the RMSF visualization from the three approaches on the PDB structure, with colors corresponding to the normalized RMSF values, indicated by the color bar on the right.

up to 500 ns on 6QNT and 6SXA and computed the RMSF obtained for five simulation durations from 100 ns to

500 ns. The RMSF obtained from the previous 30 ns simulations show high correlations with the new simulations at all five durations, as depicted in Figures S9 and S10. The correlation remains at approximately 0.8 for 6QNT, while it consistently exceeds 0.85 for 6SXA; both show no evident decrease over time, indicating that the structural fluctuations obtained from the 30 ns simulation were sufficiently stable and can serve as the foundation for our model training. Additionally, the correlations between the predicted values of RMSF-net for both 6QNT and 6SXA and the MD simulations remain above 0.8 for all five durations, demonstrating that our model genuinely learns the structural fluctuation patterns from the MD simulations.

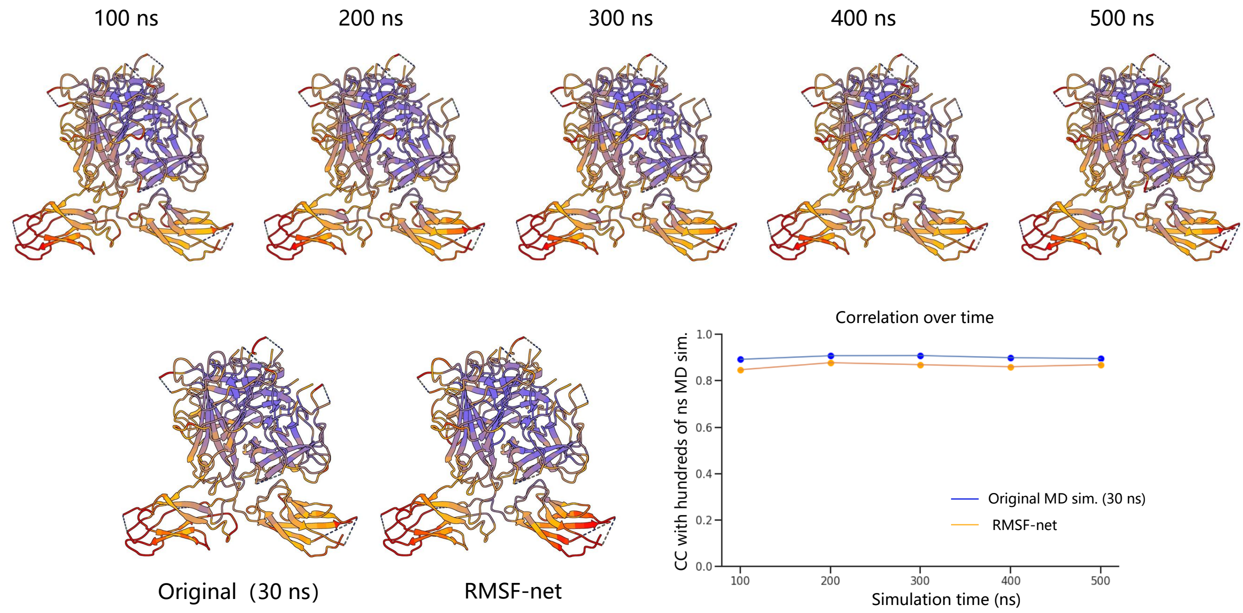

Figure S9: **Comparisons of RMSF obtained from the new several hundred nanosecond MD simulations, the original 30 ns MD simulation, and RMSF-net on 6QNT.** The top panel shows the RMSF visualizations obtained from MD simulations ranging from 100 to 500 ns on the PDB structure, with colors corresponding to the normalized RMSF values, indicated by the color bar, as in Figure S8. The bottom-left panel presents the visualizations of the original 30 ns simulation and the predicted values of RMSF-net. The correlation coefficient curves in the bottom-right panel outline the correlation between the original MD simulation and the new simulation, as well as the correlation between RMSF-net and the new MD simulation over time.

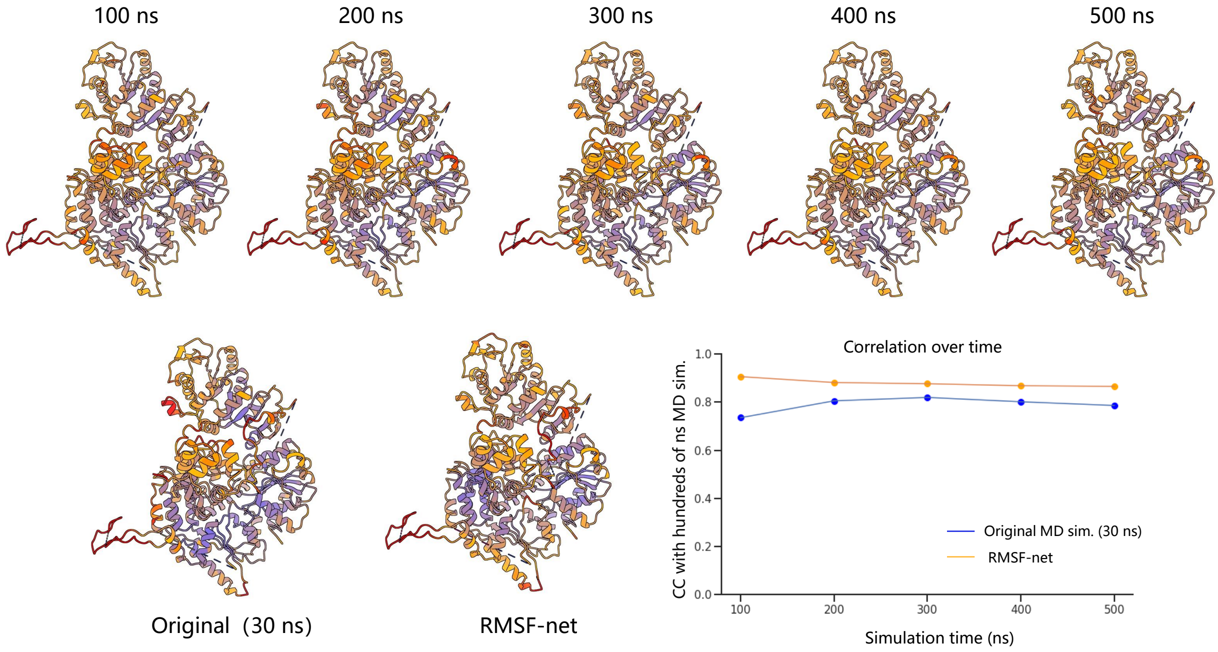

Figure S10: **Comparisons of RMSF obtained from the new several hundred nanosecond MD simulations, the original 30 ns MD simulation, and RMSF-net on 6SXA.** The top panel shows the RMSF visualizations obtained from MD simulations ranging from 100 to 500 ns on the PDB structure, with colors corresponding to the normalized RMSF values, indicated by the color bar, as in Figure S8. The bottom-left panel presents the visualizations of the original 30 ns simulation and the predicted values of RMSF-net. The correlation coefficient curves in the bottom-right panel outline the correlation between the original MD simulation and the new simulation, as well as the correlation between RMSF-net and the new MD simulation over time.

MD simulation configurations for ligand-binding proteins and membrane proteins

We selected two ligand-binding proteins (PDB 6CM9 (Morris *et al.*, 2018) and PDB 6P07 (Jones *et al.*, 2020)) to evaluate the effect of ligands on protein flexibility. The parameters used for the protein structure were AMBER ff14SB force field (Maier *et al.*, 2015). The ATP, ADP and GTP molecule parameters were taken from the AMBER parameter database (Meagher *et al.*, 2003). All other protocols were consistent with the previous ligand-free protein simulations except for the addition of position restraints to ligands during the equilibrium stages.

In addition, we conducted simulations in the membrane environment for two membrane proteins (PDB 6O1N (Dang *et al.*, 2019) and PDB 5Y4O (Yu *et al.*, 2018)). For simplicity, they were embedded in pure POPC bilayers (POPC, being the most common plasma membrane phospholipid (Marrink *et al.*, 2019)) and solvated with neutralizing ions (0.15 M NaCl) in rectangular boxes using CHARMM-GUI (Jo *et al.*, 2008). The orientation of proteins with

respect to the membrane was predicted using the PPM web server 2.0 (Lomize *et al.*, 2012). For each initial structure, MD simulation was performed using Gromacs 2022 package (Abraham *et al.*, 2015). AMBER ff14SB (Maier *et al.*, 2015) and lipid21 force field (Dickson *et al.*, 2022) were used to describe the interactions between protein structures and POPC lipid molecules. Except for the addition of position restraints to membranes during the equilibrium stages, all the other control parameters remained consistent with those of previous simulations in a membrane-free environment.

Details of the RMSF-net processing time

The time consumption of RMSF-net for dynamic inference, consisting of preprocessing, neural network inference and postprocessing, mainly exists in the first two stages. The preprocessing phase operates on the maps; hence, its time consumption is related to the map size. In contrast, the neural network inference phase acts on the data generated by preprocessing; thus, its time consumption is linked to the number of generated boxes. In filtering, all boxes whose centers contain the PDB model atoms are retained, resulting in the spatial volume of the generated box collection closely approximating the spatial volume occupied by the PDB model (referred to as the PDB size), which means that the network inference time is related to the PDB size. As depicted in Figures S8a, S9c and S9d, there is a robust linear relationship between the preprocessing time and map size (measured in terms of voxels). Additionally, preprocessing can be completed in less than 30 s for most of the maps in the dataset within 300^3 voxels. Regarding the second phase, on a CPU platform, the time for neural network processing and PDB size (in units of subboxes, i.e., k voxels) are also linearly related, as illustrated in Figures S8b and S9e. This stage can be completed within one minute for most data points where the PDB size is within 400 k voxels. If neural network inference is performed on the GPU, this stage is usually within 0.1 s, which is negligible.

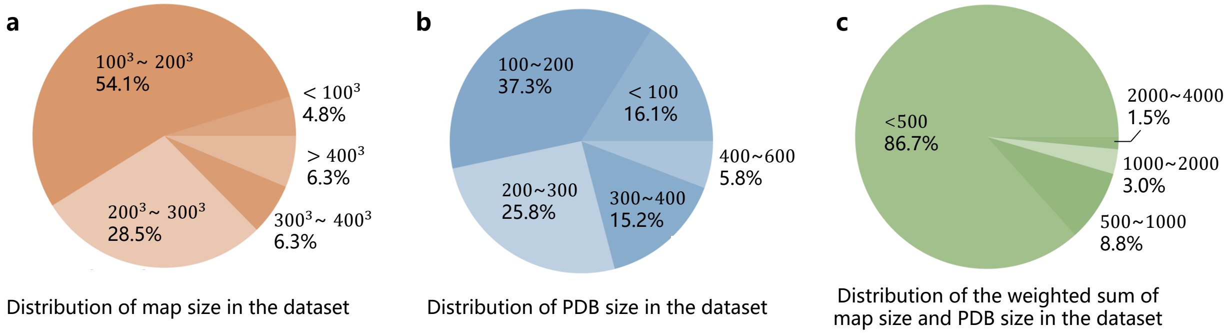

Figure S11: **Distribution of data sizes related to the RMSF-net run time across the dataset.**

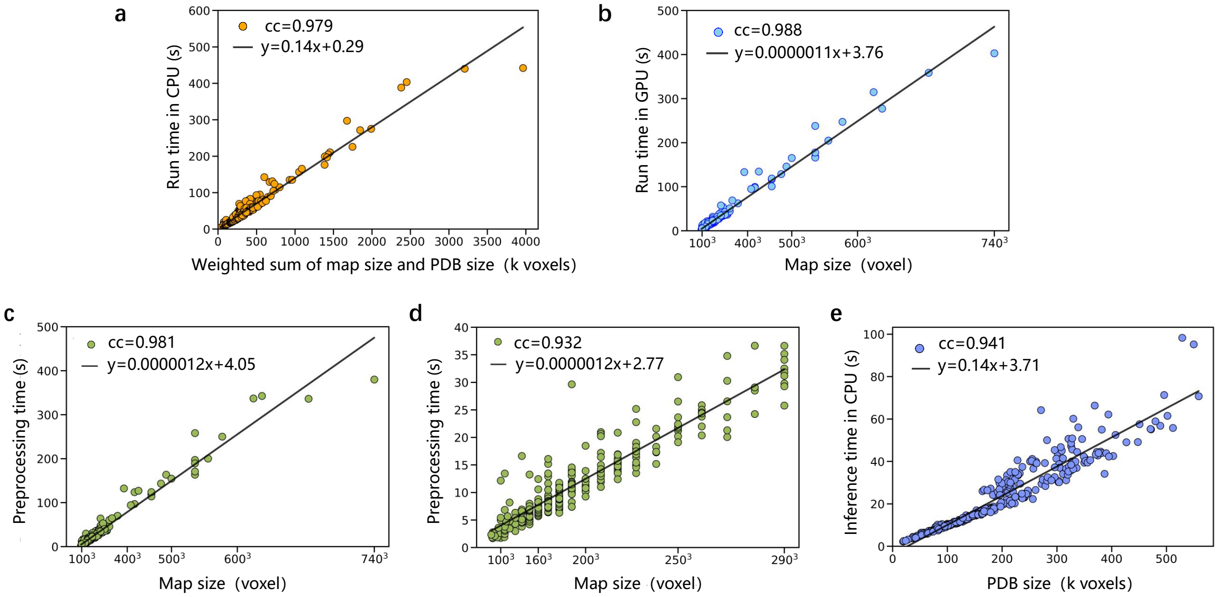

Figure S12: **Detailed relationship between the RMSF-net processing time and data size.** (a): Relationship between the RMSF-net total run time in CPUs and the weighted sum of the map size and PDB size on the dataset. The weights of the map size and PDB size obtained through linear regression are 0.0015:0.9985. (b): Relationship between the RMSF-net total run time on GPUs and the map size on the dataset. (c): Relationship between the RMSF-net preprocessing time and the map size on the dataset. (d): Relationship between the RMSF-net preprocessing time and the map size for the data points for which the map size is less than 300^3. (e): Relationship between the RMSF-net network inference time on CPUs and the PDB size on the dataset.

Table S1: **Data processing of RMSF-net on 10 data entries**

Data entry	Data size		
	map file_size(MB)	selected_boxes	total_boxes
3J63/EMD-5830	3.82	187	512
5K0Z/EMD-8191	22.25	133	216
3J89/EMD-6123	3.82	85	343
5K10/EMD-8192	30.52	102	343
6J6J/EMD-0689	8	69	729
6GGS/EMD-4399	3.82	105	729
3J9E/EMD-6240	9.02	78	800
6S59/EMD-10099	13.95	232	810
3J9D/EMD-6239	11.76	99	990
6PP5/EMD-20419	27	257	3375

Table S2: **Computational cost of RMSF-net on 10 data entries**

Data entry	Run time in CPU (s)			Run time in GPU (s)			generated data file size (MB)
	preprocessing	model inference & postprocessing	total	preprocessing	model inference & postprocessing	total	
3J63/EMD-5830	2.08	17.23	19.31	2.22	0.27	2.49	92
5K0Z/EMD-8191	2.33	12.33	14.66	2.52	0.19	2.71	65
3J89/EMD-6123	1.69	8.30	9.98	2.15	0.13	2.28	42
5K10/EMD-8192	2.76	9.68	12.44	2.66	0.16	2.82	50
6J6J/EMD-0689	2.05	6.30	8.35	2.24	0.11	2.35	34
6GGS/EMD-4399	1.84	9.65	11.48	2.01	0.16	2.18	52
3J9E/EMD-6240	1.99	7.60	9.59	2.26	0.13	2.39	39
6S59/EMD-10099	3.58	33.92	37.50	3.21	0.31	3.52	114
3J9D/EMD-6239	2.63	8.57	11.20	2.65	0.16	2.81	49
6PP5/EMD-20419	7.50	24.60	32.10	6.93	0.38	7.30	126

Table S3: **Computational cost of DEFMap on 10 data entries**

Data entry	Run time on CPUs (min)			Run time on GPUs (min)			generated data file size (GB)
	preprocessing	model inference	total	preprocessing	model inference	total	
3J63/EMD-5830	1.55	10.10	11.65	0.48	24.92	25.40	2.9
5K0Z/EMD-8191	5.63	68.67	74.30	5.50	41.18	46.68	19
3J89/EMD-6123	1.08	10.12	11.20	1.50	24.72	26.22	2.9
5K10/EMD-8192	7.82	99.53	107.35	8.00	50.70	58.70	27
6J6J/EMD-0689	2.32	23.47	25.78	2.22	28.73	30.95	6.4
6GGS/EMD-4399	0.50	10.60	11.10	0.85	25.17	26.02	2.9
3J9E/EMD-6240	3.18	38.70	41.88	2.95	32.72	35.67	11
6S59/EMD-10099	4.60	54.50	59.10	4.83	37.63	42.47	15
3J9D/EMD-6239	3.05	32.48	35.53	3.07	32.85	35.92	9.7
6PP5/EMD-20419	10.50	71.00	81.50	9.10	38.00	47.10	23

Accessible and user-friendly RMSF-net

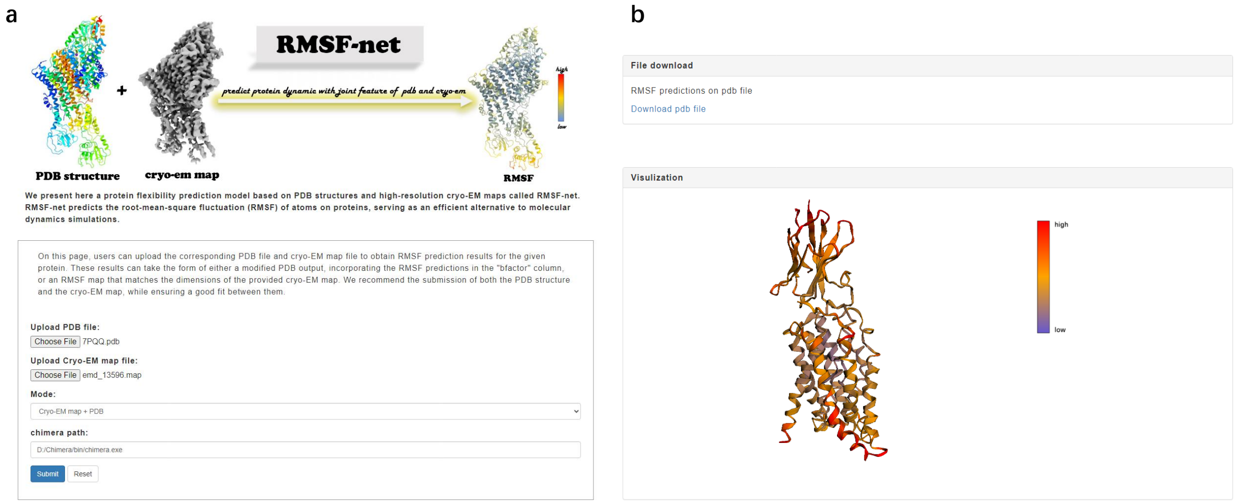

Figure S13: **Web pages of RMSF-net.** (a): The introduction and submission page. (b): The results page.

To facilitate convenient usage of RMSF-net, we have developed it as a web-based software with a user-friendly web front-end built on the Django framework. Users can easily install RMSF-net by running the provided executable (exe) file (available for download from Figshare archive https://doi.org/10.6084/m9.figshare.25893670) and utilize their local computing resources (CPUs or GPUs) for RMSF predictions. The only prerequisite for executing RMSF-net functions is to have UCSF Chimera (Pettersen *et al.*, 2004) installed from http://www.cgl.ucsf.edu/chimera/.

After installation, opening the entry file will launch the RMSF-net web page in the local browser, as shown in Figure S13a. On this page, users can submit RMSF prediction tasks for their proteins of interest by uploading the corresponding cryo-EM map and PDB file, along with specifying the path to the Chimera executable. After a few seconds or minutes of inference, the results page will be generated and displayed. Figure S13b depicts an example of the results page, which consists of two sections. The first section, named ‘File Download’, allows users to download the predicted RMSF values mapped onto the B-factor column of the PDB file. The second section, ‘Visualization’, employs 3DMol.js (Rego and Koes, 2015) to present the protein in a color-coded cartoon format based on the predicted RMSF for residues. Corresponding to the three models outlined in this study, three options are available: ‘only PDB’, ‘only cryo-EM map’, and ‘both’. Users can select different options to review the prediction results.

References

Abraham, M. J., Murtola, T., Schulz, R., Páll, S., Smith, J. C., Hess, B., and Lindahl, E. (2015). Gromacs: High performance molecular simulations through multi-level parallelism from laptops to supercomputers. *SoftwareX*, **1**, 19–25.

Dang, S., Van Goor, M. K., Asarnow, D., Wang, Y., Julius, D., Cheng, Y., and van der Wijst, J. (2019). Structural insight into trpv5 channel function and modulation. *Proceedings of the National Academy of Sciences*, **116**(18), 8869–8878.

Dickson, C. J., Walker, R. C., and Gould, I. R. (2022). Lipid21: complex lipid membrane simulations with amber. *Journal of chemical theory and computation*, **18**(3), 1726–1736.

He, J. and Huang, S.-Y. (2021). EMNUSS: a deep learning framework for secondary structure annotation in cryo-EM maps. *Briefings in Bioinformatics*, **22**(6). bbab156.

Jo, S., Kim, T., Iyer, V. G., and Im, W. (2008). Charmm-gui: a web-based graphical user interface for charmm. *Journal of computational chemistry*, **29**(11), 1859–1865.

Jones, M., Beuron, F., Borg, A., Nans, A., Earl, C. P., Briggs, D. C., Snijders, A. P., Bowles, M., Morris, E. P., Linch, M., *et al.* (2020). Cryo-em structures of the xpf-ercc1 endonuclease reveal how dna-junction engagement disrupts an auto-inhibited conformation. *Nature communications*, **11**(1), 1120.

Lomize, M. A., Pogozheva, I. D., Joo, H., Mosberg, H. I., and Lomize, A. L. (2012). Opm database and ppm web server: resources for positioning of proteins in membranes. *Nucleic acids research*, **40**(D1), D370–D376.

Maier, J. A., Martinez, C., Kasavajhala, K., Wickstrom, L., Hauser, K. E., and Simmerling, C. (2015). ff14sb: improving the accuracy of protein side chain and backbone parameters from ff99sb. *Journal of chemical theory and computation*, **11**(8), 3696–3713.

Marrink, S. J., Corradi, V., Souza, P. C., Ingolfsson, H. I., Tieleman, D. P., and Sansom, M. S. (2019). Computational modeling of realistic cell membranes. *Chemical reviews*, **119**(9), 6184–6226.

Meagher, K. L., Redman, L. T., and Carlson, H. A. (2003). Development of polyphosphate parameters for use with the amber force field. *Journal of computational chemistry*, **24**(9), 1016–1025.

Morris, K. L., Buffalo, C. Z., Stürzel, C. M., Heusinger, E., Kirchhoff, F., Ren, X., and Hurley, J. H. (2018). Hiv-1 nefs are cargo-sensitive ap-1 trimerization switches in tetherin downregulation. *Cell*, **174**(3), 659–671.

Mostosi, P., Schindelin, H., Kollmannsberger, P., and Thorn, A. (2020). Haruspex: A neural network for the automatic identification of oligonucleotides and protein secondary structure in cryo-electron microscopy maps. *Angewandte Chemie International Edition*, **59**(35), 14788–14795.

Pettersen, E. F., Goddard, T. D., Huang, C. C., Couch, G. S., Greenblatt, D. M., Meng, E. C., and Ferrin, T. E. (2004). Ucsf chimera—a visualization system for exploratory research and analysis. *Journal of computational chemistry*, **25**(13), 1605–1612.

Rego, N. and Koes, D. (2015). 3dmol. js: molecular visualization with webgl. *Bioinformatics*, **31**(8), 1322–1324.

Vassal-Stermann, E., Effantin, G., Zubieta, C., Burmeister, W., Iseni, F., Wang, H., Lieber, A., Schoehn, G., and Fender, P. (2019). Cryoem structure of adenovirus type 3 fibre with desmoglein 2 shows an unusual mode of receptor engagement. *Nature communications*, **10**(1), 1181.

Walls, A. C., Tortorici, M. A., Bosch, B.-J., Frenz, B., Rottier, P. J., DiMaio, F., Rey, F. A., and Veesler, D. (2016). Cryo-electron microscopy structure of a coronavirus spike glycoprotein trimer. *Nature*, **531**(7592), 114–117.

Yu, J., Zhang, B., Zhang, Y., Xu, C.-q., Zhuo, W., Ge, J., Li, J., Gao, N., Li, Y., and Yang, M. (2018). A binding-block ion selective mechanism revealed by a na/k selective channel. *Protein & cell*, **9**(7), 629–639.
