# Supplementary material for: Accurate Prediction of Protein Structural Flexibility by Deep Learning Integrating Intricate Atomic Structures and Cryo-EM Density Information
Source: Nat Commun. 2024 Jul 2;15:5538. doi: 10.1038/s41467-024-49858-x (PMC11219796; doi:10.1038/s41467-024-49858-x)
Supplement: Supplementary file 3 — Reporting Summary [file 41467_2024_49858_MOESM3_ESM.pdf]

nature portfolio

Corresponding author(s): Renmin Han

Last updated by author(s): May 25, 2024

Reporting Summary

Nature Portfolio wishes to improve the reproducibility of the work that we publish. This form provides structure for consistency and transparency in reporting. For further information on Nature Portfolio policies, see our Editorial Policies and the Editorial Policy Checklist.

Statistics

For all statistical analyses, confirm that the following items are present in the figure legend, table legend, main text, or Methods section.

n/a	Confirmed	
☐	☒	The exact sample size (*n*) for each experimental group/condition, given as a discrete number and unit of measurement
☒	☐	A statement on whether measurements were taken from distinct samples or whether the same sample was measured repeatedly
☒	☐	The statistical test(s) used AND whether they are one- or two-sided<br>*Only common tests should be described solely by name; describe more complex techniques in the Methods section.*
☒	☐	A description of all covariates tested
☒	☐	A description of any assumptions or corrections, such as tests of normality and adjustment for multiple comparisons
☐	☒	A full description of the statistical parameters including central tendency (e.g. means) or other basic estimates (e.g. regression coefficient) AND variation (e.g. standard deviation) or associated estimates of uncertainty (e.g. confidence intervals)
☒	☐	For null hypothesis testing, the test statistic (e.g. *F*, *t*, *r*) with confidence intervals, effect sizes, degrees of freedom and *P* value noted<br>*Give P values as exact values whenever suitable.*
☒	☐	For Bayesian analysis, information on the choice of priors and Markov chain Monte Carlo settings
☒	☐	For hierarchical and complex designs, identification of the appropriate level for tests and full reporting of outcomes
☒	☐	Estimates of effect sizes (e.g. Cohen's *d*, Pearson's *r*), indicating how they were calculated

Our web collection on statistics for biologists contains articles on many of the points above.

Software and code

Policy information about availability of computer code

Data collection	All data used is from the public EMDB and PDB data repository. No software was used for data collection.
Data analysis	The method RMSF-net source code can be downloaded from https://github.com/XintSong/RMSF-net. The visualization of density maps and PDB atomic models was performed in UCSF ChimeraX (1.7) and PyMOL (2.5.3). We also used UCSF Chimera(1.16) to convert PDB models to density maps.

For manuscripts utilizing custom algorithms or software that are central to the research but not yet described in published literature, software must be made available to editors and reviewers. We strongly encourage code deposition in a community repository (e.g. GitHub). See the Nature Portfolio guidelines for submitting code & software for further information.

Data

Policy information about availability of data

All manuscripts must include a data availability statement. This statement should provide the following information, where applicable:

- Accession codes, unique identifiers, or web links for publicly available datasets
- A description of any restrictions on data availability
- For clinical datasets or third party data, please ensure that the statement adheres to our policy

The data used as examples are available from the EMDB and PDB (accession codes specified in the main text and figure captions). The full list of the dataset can be

found in the Source Data files. The MD simulation annotations for RMSF-net training and evaluation can be download from Figshare (https://doi.org/10.6084/m9.figshare.25893670).

Research involving human participants, their data, or biological material

Policy information about studies with human participants or human data. See also policy information about sex, gender (identity/presentation), and sexual orientation and race, ethnicity and racism.

Reporting on sex and gender	N/A
Reporting on race, ethnicity, or other socially relevant groupings	N/A
Population characteristics	N/A
Recruitment	N/A
Ethics oversight	N/A

Note that full information on the approval of the study protocol must also be provided in the manuscript.

Field-specific reporting

Please select the one below that is the best fit for your research. If you are not sure, read the appropriate sections before making your selection.

☒ Life sciences ☐ Behavioural & social sciences ☐ Ecological, evolutionary & environmental sciences

For a reference copy of the document with all sections, see nature.com/documents/nr-reporting-summary-flat.pdf

Life sciences study design

All studies must disclose on these points even when the disclosure is negative.

Sample size	Our data were collected from two sources, the high-resolution (2-4 Å) cryo-EM map dataset in EMNUSS, and the high-resolution (2-4 Å) cryo-EM structure data from October 2020 to November 2022 in EMDB and PDB. The final sample size was 335, determined by data screening and molecular dynamics simulation results.
Data exclusions	Data that does not meet the following criteria is excluded: map resolution between 2-4 Å, cryo-EM map and PDB model with a fitness above 0.7, containing at least one alpha helix or beta strand, with no missing chains or nucleic acids, and successfully underwent Molecular Dynamics simulation. The reason is that our study targets typical proteins, along with their high-resolution cryo-EM maps and corresponding PDB structures, and necessitates the successful acquisition of RMSF through MD simulations.
Replication	The results can be replicated using the same dataset and annotated data files we provide. If all attempts at replication are successful. Our code is open-source on Github (https://github.com/XintSong/RMSF-net), and the replication can be verified.
Randomization	During five-fold cross-validation of the model, the dataset is randomly divided into five subsets.
Blinding	The investigators were blinded to group allocation.

Reporting for specific materials, systems and methods

We require information from authors about some types of materials, experimental systems and methods used in many studies. Here, indicate whether each material, system or method listed is relevant to your study. If you are not sure if a list item applies to your research, read the appropriate section before selecting a response.

Materials & experimental systems

n/a	Involved in the study	
☒	☐	Antibodies
☒	☐	Eukaryotic cell lines
☒	☐	Palaeontology and archaeology
☒	☐	Animals and other organisms
☒	☐	Clinical data
☒	☐	Dual use research of concern
☒	☐	Plants

Methods

n/a	Involved in the study	
☒	☐	ChIP-seq
☒	☐	Flow cytometry
☒	☐	MRI-based neuroimaging

Plants

Seed stocks	*Report on the source of all seed stocks or other plant material used. If applicable, state the seed stock centre and catalogue number. If plant specimens were collected from the field, describe the collection location, date and sampling procedures.*
Novel plant genotypes	*Describe the methods by which all novel plant genotypes were produced. This includes those generated by transgenic approaches, gene editing, chemical/radiation-based mutagenesis and hybridization. For transgenic lines, describe the transformation method, the number of independent lines analyzed and the generation upon which experiments were performed. For gene-edited lines, describe the editor used, the endogenous sequence targeted for editing, the targeting guide RNA sequence (if applicable) and how the editor was applied.*
Authentication	*Describe any authentication procedures for each seed stock used or novel genotype generated. Describe any experiments used to assess the effect of a mutation and, where applicable, how potential secondary effects (e.g. second site T-DNA insertions, mosiacism, off-target gene editing) were examined.*
